# Identification of a set of KSRP target transcripts upregulated by PI3K-AKT signaling

**DOI:** 10.1186/1471-2199-8-28

**Published:** 2007-04-16

**Authors:** Tina Ruggiero, Michele Trabucchi, Marco Ponassi, Giorgio Corte, Ching-Yi Chen, Latifa al-Haj, Khalid SA Khabar, Paola Briata, Roberto Gherzi

**Affiliations:** 1 Istituto Nazionale per la Ricerca sul Cancro (IST), 16132 Genova, Italy; 2 DOBIG, University of Genova, 16132 Genova, Italy; 3 Department of Biochemistry and Molecular Genetics, University of Alabama at Birmingham, Birmingham, AL 35294, USA; 4 Program in Biomolecular Research, King Faisal Specialist Hospital and Research Center, Riyadh 11211, Saudi Arabia

## Abstract

**Background:**

KSRP is a AU-rich element (ARE) binding protein that causes decay of select sets of transcripts in different cell types. We have recently described that phosphatidylinositol 3-kinase/AKT (PI3K-AKT) activation induces stabilization and accumulation of the labile β-catenin mRNA through an impairment of KSRP function.

**Results:**

Aim of this study was to identify additional KSRP targets whose stability and steady-state levels are enhanced by PI3K-AKT activation. First, through microarray analyses of the AU-rich transcriptome in pituitary αT3-1 cells, we identified 34 ARE-containing transcripts upregulated in cells expressing a constitutively active form of AKT1. In parallel, by an affinity chromatography-based technique followed by microarray analyses, 12 mRNAs target of KSRP, additional to β-catenin, were identified. Among them, seven mRNAs were upregulated in cells expressing activated AKT1. Both steady-state levels and stability of these new KSRP targets were consistently increased by either KSRP knock-down or PI3K-AKT activation.

**Conclusion:**

Our study identified a set of transcripts that are targets of KSRP and whose expression is increased by PI3K-AKT activation. These mRNAs encode RNA binding proteins, signaling molecules and a replication-independent histone. The increased expression of these gene products upon PI3K-AKT activation could play a role in the cellular events initiated by this signaling pathway.

## Background

Regulated mRNA decay is a key factor in determining the expression pattern of many genes including those encoding for cytokines, proto-oncogenes, cell cycle regulators, and growth factors [1]. Adenylate-uridylate-rich elements (AREs), present in the 3'-untranslated region (3'UTR) of many inherently labile mRNAs, are the most widespread and best characterized destabilizing sequences [1,2]. Impairment of the ARE-mediated mRNA decay results in abnormal cell proliferation and angiogenesis, leading to cancer insurgence and progression [3], as well as in inflammatory diseases such as Crohn-like inflammatory bowel disease and inflammatory arthritis [4].

The interaction of regulatory proteins, ARE-binding proteins (ARE-BPs), with their target labile mRNAs determines the half-life (t1/2) of these transcripts. Some ARE-BPs are decay-promoting factors (TTP, BRF1, KSRP) [1]. Others, such as HuR, are stabilizing factors, whereas AUF1 mainly promotes decay although certain isoforms might be stabilizers of ARE-containing mRNAs [1,5,6]. According to the recently proposed recruitment model, destabilizing ARE-BPs, recruit the enzymatic degradation machinery to their target mRNAs [7-9].

We and others have recently reported that KSRP promotes rapid decay of several ARE-containing mRNAs both in vitro and in vivo and that extracellular stimuli regulate its activity [7,10-13]. We have shown that activation of either Wnt/β-catenin pathway in αT3-1 cells [10] or MAPK p38 signaling in C2C12 myoblasts [11] selectively regulates the stability of specific sets of labile mRNAs targeting KSRP. More recently, we demonstrated that phosphatidylinositol 3-kinase/AKT (PI3K-AKT) signaling activation induces stabilization and enhances the steady-state levels of β-catenin mRNA in pituitary αT3-1 cell line through phosphorylation and functional inactivation of KSRP [12]. PI3K-AKT signaling exerts a central role in metabolism, cell survival, motility, transcription and cell-cycle progression [[12] and literature cited therein].

It has been recently suggested that control of mRNA decay is utilized by the cell to coordinate the expression of genes involved in specific processes leading to the notion of 'post-transcriptional operons' [14]. This would allow multiple genes to be co-regulated by a similar array of RNA-binding proteins in response to certain stimuli. On this basis, we hypothesized that PI3K-AKT activation could regulate the expression of transcripts additional to β-catenin by targeting KSRP. According to this hypothesis, a subset of KSRP target transcripts should be stabilized in response to PI3K-AKT signaling.

To verify this hypothesis, we systematically searched, among the AU-rich transcriptome, for KSRP target transcripts whose expression was upregulated by PI3K-AKT signaling. We identified a set of labile mRNAs, stabilized upon either KSRP knock-down or PI3K-AKT activation, encoding signaling factors, RNA binding proteins, and a replication-independent histone. These proteins could play a role in the cascade of cellular events initiated by PI3K-AKT activation.

## Results

### Identification of KSRP target transcripts upregulated in cells expressing constitutively active myrAKT1

We recently found by microarray analyses of the AU-rich transcriptome that β-catenin mRNA is stabilized and upregulated by PI3K-AKT signaling in αT3-1 cells in a KSRP-regulated manner [12]. To systematically search for inherently labile transcripts whose expression is induced by PI3K-AKT activation, we compared RNA expression profiles of mock-transfected αT3-1 cells (mock-αT3-1) to those of αT3-1 cells expressing a constitutively active AKT1 (αT3-1-myrAKT1 [12]) using the AU-rich element-based cDNA microarrays [15]. This array system contains approximately 2500 cDNA probes for ARE-containing mRNAs and over 1000 cDNA probes for non-ARE mRNAs and control housekeeping genes (Additional file 1). For each cell line we obtained expression profiles from two independent RNA samples. As shown in Table 1, we identified 35 transcripts at least twofold overrepresented in αT3-1-myrAKT1 when compared to mock-αT3-1 cells. Among these, β-catenin mRNA has been previously reported and characterized (see above, [12]). All the identified transcripts display AREs in their 3'UTR and encode proteins belonging to distinct functional categories (Table 1).

In parallel, in order to identify mRNA targets of KSRP in αT3-1 cells, we adopted the SNAAP technique (isolation of specific nucleic acids associated with proteins) developed by Kiledjian and coworkers [16] using GST-fused KSRP as the affinity chromatography matrix. This technique allows to isolate only those mRNAs for which the fusion protein of interest has a high affinity at the physiological salt concentrations in the context of a ribonucleoprotein complex [16]. The identification of KSRP-bound mRNAs was performed by screening the AU-rich element-based cDNA microarrays [15]. Sequence analysis performed on 314 KSRP-interacting mRNAs identified by SNAAP (transcripts upregulated by at least 1.8-fold in KSRP-bound samples), demonstrated over-representation of ARE motifs when compared to 314 non-KSRP target transcripts (Additional file 2). Eighty genes whose mRNAs interacted with KSRP (log_2 _ratio > 1.5, see Additional file 3) were identified. Thirteen of these mRNAs (including previously characterized β-catenin mRNA [12]) displayed a more than 3 fold enrichment (our arbitrary cutoff) over the control GST matrix and were considered for further analysis (Table 2). In order to select KSRP target transcripts whose expression is induced by PI3K-AKT activation, we performed a comparative analysis of the results of the two screenings. We sorted out seven unanticipated mRNAs that were both enriched upon SNAAP isolation and over-represented in αT3-1-myrAKT1 cells (typed in bold in Table 1). These transcripts encode three distinct RNA binding proteins, hnRNPA1, hnRNPF, and hnRNPA/B, three proteins implicated in cell signaling, the alpha stimulating subunit of guanine nucleotide binding protein (Gsα, encoded by the GNAS locus, GNAS), the alpha isoform of the catalytic subunit of the protein phosphatase 2 (PP2ACA), and the SH domain containing protein sorbin (SORBIN), as well as the replication-independent histone H3.3A (H3.3A).

**Figure 1:**
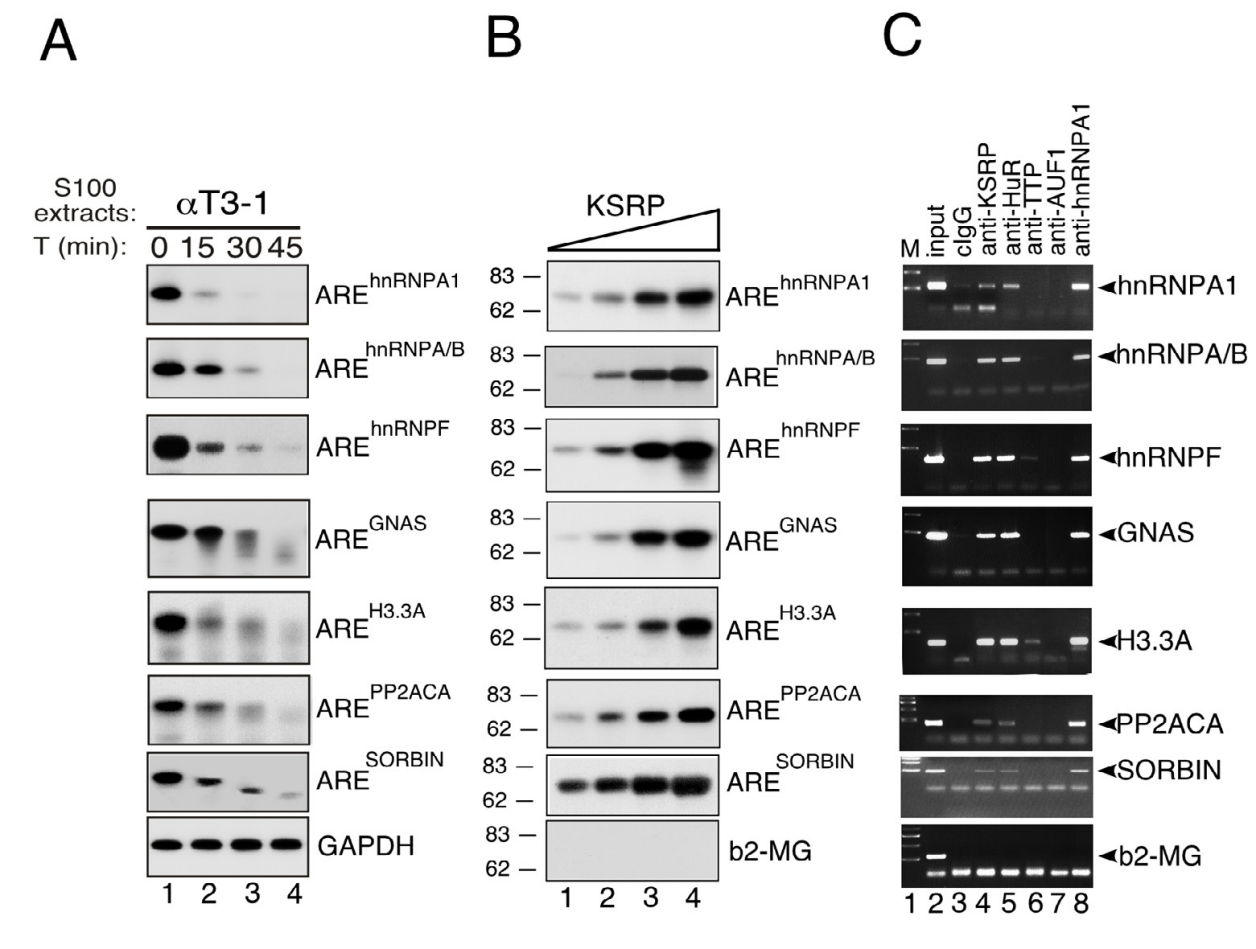
KSRP associates with a set of unstable mRNAs overrepresented in myrAKT1-αT3-1 cells. (A) In vitro RNA degradation assays using S100 extracts from αT3-1 cells. Internally ^32^P-labeled, capped RNA substrates (see Additional file 4 for sequences) were incubated with extracts for the indicated times and their decay analyzed as described in Methods. (B) The interaction between ^32^P-labeled RNAs (indicated on the right) and recombinant purified KSRP (30–300 nM) was evaluated by UV-crosslinking. (C) Immunoprecipitation of ribonucleoprotein complexes containing different KSRP target mRNAs. The proteins were immunoprecipitated from αT3-1 cell extracts using the indicated antibodies. RNA was extracted from the immune complexes and analyzed by RT-PCR as described in Methods.

The ARE-containing regions of the novel KSRP target transcripts (Additional file 4) displayed a potent destabilizing function both in vitro (Figure 1A) and in intact cells (see below, Figure 3C). Purified recombinant KSRP was able to bind, in a dose-response manner, to the AREs of the novel KSRP targets in vitro (Figure 1B). To validate the interaction of the endogenous KSRP with its target transcripts, we performed immunoprecipitation experiments of ribonucleoprotein complexes in αT3-1 cells. As shown in Figure 1C, the seven transcripts were immunoprecipitated by anti-KSRP antibody as well as by anti-hnRNPA1 antibody. No KSRP target mRNA was detected in anti-AUF1 immunoprecipitates under standard experimental conditions (Figure 1C). However, when the amount of cDNAs obtained by retrotranscription and used in PCR reactions was increased by 10-fold, bands corresponding to hnRNPA/B and prothymosin α (PTMA) mRNAs (both already identified as AUF1 targets [17]) were detected (Additional file 5). Interestingly, also anti-HuR antibody immunoprecipitated the KSRP target mRNAs while anti-TTP antibody immunoprecipitated only hnRNPF and GNAS (Figure 1C) as well as GM-CSF mRNA which is a typical TTP target transcript (data not shown). We obtained similar results performing immunoprecipitation of ribonucleoprotein complexes in C2C12 myoblasts (data not shown).

### KSRP associates with AUF1p45 and hnRNPA1 in the cytoplasm of αT3-1 cells

**Figure 2:**
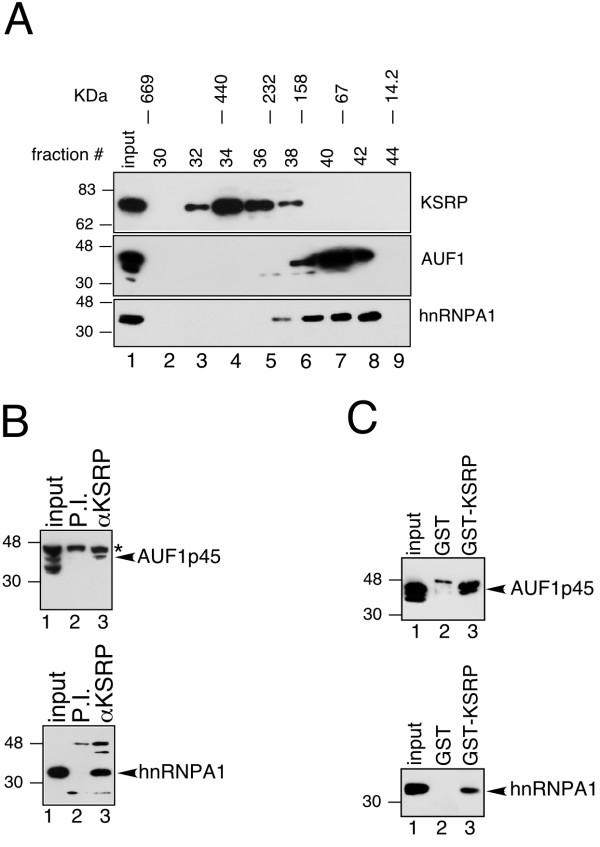
KSRP associates with AUF1p45 and hnRNPA1 in cytoplasmic extracts of aT3-1 cells. (A) S100 extracts from αT3-1 cells were subjected to gel filtration chromatography on a Superose 6 column. Aliquots of the eluted fractions were analyzed by Western blotting using the indicated antibodies. (B) RNase A-treated S100 extracts from αT3-1 cells were immunoprecipitated with preimmune (lane 2) or anti-KSRP (lane 3) sera and analyzed by immunoblotting with either anti-AUF1 (top) or anti-HnRNPA1 (bottom) antibodies. The arrows mark the position of either AUF1p45 or hnRNPA1, while the asterisk marks a anti-AUF1 cross-reacting band. (C) GST-pulldown of either endogenous AUF1p45 (top) or endogenous hnRNPA1 (bottom) from S100 extracts of αT3-1 cells using either control GST or GST-KSRP. Proteins were analyzed by immunoblotting using the indicated antibodies. The arrows mark the position of either AUF1p45 or hnRNPA1.

HPLC gel filtration of S100 extracts from αT3-1 cells followed by anti-KSRP immunoblotting analysis, showed that KSRP is present in fractions of molecular mass ranging from 150 KDa to over-440 KDa (Figure 2A, top panel). We have previously demonstrated that KSRP functionally associates with components of the mRNA decay machinery [7,9,18]. In order to identify KSRP molecular partners additional to those already known, a yeast two-hybrid screening using KSRP as a bait was performed. We identified several potential KSRP interacting proteins (listed in Table 3) including the chaperone protein 14-3-3ζ whose function in KSRP-dependent β-catenin mRNA decay was recently described by Gherzi et al. [12]. We found, among others, the cDNAs encoding two *bona fide *ARE binding proteins, the p45 isoform of AUF1 (AUF1p45) [6] and hnRNPA1 [19]. Both AUF1p45 and hnRNPA1 elute together with KSRP in gel filtration fractions ranging from 100 to 200 KDa (Figure 2A, middle and bottom panels and data not shown). The interaction of either AUF1p45 or hnRNPA1 with KSRP was confirmed by anti-KSRP immunoprecipitation of RNase A-treated αT3-1 cytoplasmic extracts followed by either anti-AUF1 or anti-hnRNPA1 immunoblotting (Figure 2B). GST-fused KSRP was able to pull-down both endogenous AUF1p45 and hnRNPA1 from αT3-1 cytoplasmic extracts (Figure 2C). These data, together with those presented in Figure 1C suggest that KSRP target transcripts belong to a ribonucleoprotein complex including AUF1p45 and hnRNPA1.

We investigated whether either AUF1p45 or hnRNPA1 or both directly interacted with KSRP target transcripts. UV-crosslinking experiments failed to display high affinity interaction of these ARE-BPs with the KSRP target transcripts in vitro (data not shown). This finding suggest that AUF1p45 and hnRNPA1 are part of the KSRP-containing ribonucleoprotein complex but do not directly interact with KSRP targets.

### KSRP knock-down in αT3-1 cells stabilizes KSRP target transcripts

**Figure 3:**
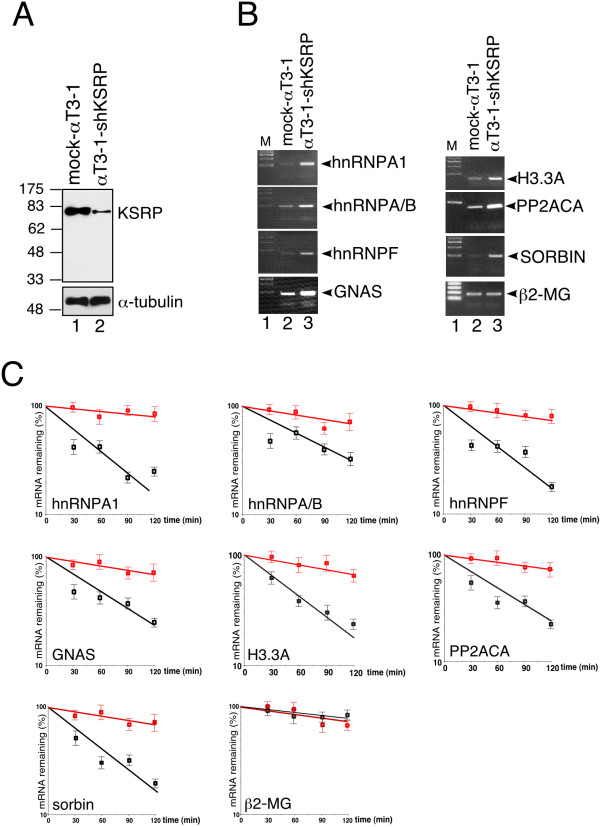
KSRP is required for rapid degradation of a set of unstabletranscripts. (A) Immunoblot analysis of total extracts from either mock-αT3-1 (empty pSUPER-Puro vector-transfected) or αT3-1-shKSRP (pSUPER-Puro-shKSRP-transfected) cells using affinity-purified anti-KSRP and anti-α-tubulin antibodies. (B) Expression of a set of KSRP-interacting mRNAs and β2-MG (as a control), monitored by RT-PCR, in either mock-αT3-1, or αT3-1-shKSRP cells. (C) Semi quantitative RT-PCR analysis of both labile KSRP-associated mRNAs and β2-MG mRNA in either mock-αT3-1 (black lines), or αT3-1-shKSRP cells (red lines). Total RNA was isolated at the indicated times after addition of Actinomycin D. The amount of each transcript was quantitated by densitometry and plotted using a linear regression program. The values shown are averages (± SEM) of three independent experiments performed in duplicates. A quantitation of the transcripts' t(1/2) is presented in Additional file 6.

To verify the relevance of KSRP in the decay control of its target transcripts, stable knock-down of KSRP using a short-hairpin vector was performed in αT3-1 cells (αT3-1-shKSRP; Figure 3A). KSRP knock-down led to two- to five-fold increase of the steady-state levels of KSRP target mRNAs in αT3-1-shKSRP when compared to mock-transfected cells (Figure 3B). No changes were seen with the control β2-MG RNA levels (Figure 3B). Next, using actinomycin D, we analyzed the t1/2 of the identified KSRP target mRNAs in both mock-αT3-1 and αT3-1-shKSRP cells. Results presented in Figure 3C showed that KSRP knock-down in αT3-1 cells strongly increased the t1/2 of all the transcripts (from less than 60 min. in mock-αT3-1 to more than 2 hours in αT3-1-shKSRP cells, see Additional file 6).

Overall these data indicate that KSRP interacts with a subset of mRNAs up-regulated in cells expressing constitutively active AKT1 and regulates their stability and steady-state levels in αT3-1 cells.

### PI3K-AKT activation stabilizes KSRP target transcripts

**Figure 4:**
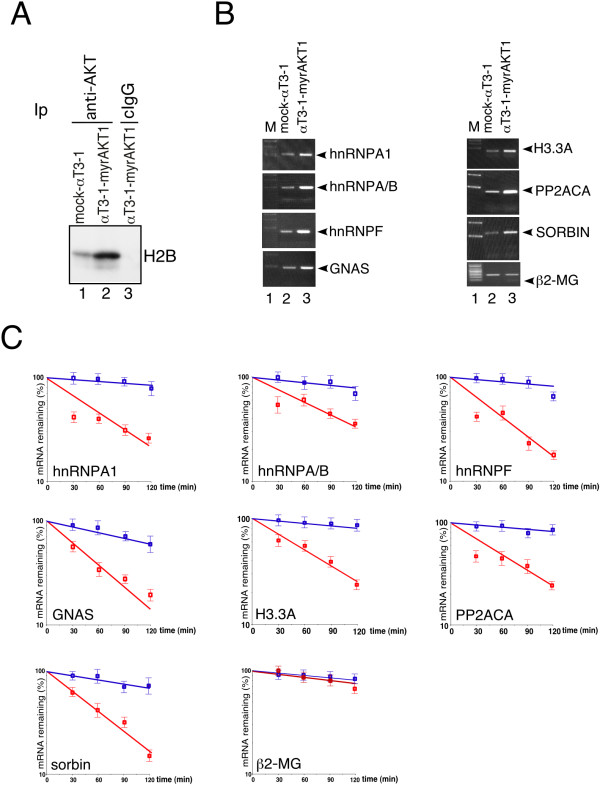
PI3K-AKT signaling stabilizes a set of KSRP-interacting mRNAs and increases their expression. (A) Either mock-αT3-1 or αT3-1-myrAKT1 cells were lysed and total extracts were immunoprecipitated (Ip) with either anti-AKT antibody or control IgG (cIgG). Pellets were incubated (20 min at 30°C) with histone 2B (H2B) in kinase buffer in the presence of γ[^32^P]ATP under gentle shaking. Labeled proteins were separated by SDS-PAGE and detected by autoradiography. (B) Expression of KSRP-interacting mRNAs and β2-MG (control transcript), monitored by RT-PCR, in either mock-αT3-1 or αT3-1-myrAKT1 cells. (C) Semi quantitative RT-PCR analysis of both KSRP-interacting mRNAs and β2-MG (control transcript) in either mock-αT3-1 (red lines) or αT3-1-myrAKT1 (blue lines). Total RNA was isolated at the indicated times after addition of Actinomycin D. The amount of each transcript was quantitated by densitometry and plotted using a linear regression program. The values shown are averages (± SEM) of three independent experiments performed in duplicates. A quantitation of the transcripts' t(1/2) is presented in Additional file 7.

We recently showed that PI3K-AKT activation in αT3-1 cells stabilizes β-catenin mRNA and induces its accumulation [12]. These events are mediated by KSRP phosphorylation and functional inactivation [12]. To investigate whether the activation of the pathway affects the stability of the novel KSRP targets, we took advantage of αT3-1-myrAKT1 cells [12]. As shown in Figure 4A, the kinase activity immunoprecipitated with anti-AKT antibody was ~5 fold higher in αT3-1-myrAKT1 than in mock-αT3-1 cells thus demonstrating that active AKT kinase was present in αT3-1 cells expressing myrAKT1. The steady-state levels of KSRP target mRNAs were increased by 2.5-4 fold in intact αT3-1-myrAKT1 cells (Figures 4B). In addition, the t1/2 of the KSRP mRNA targets was prolonged above 2 hours as a result of AKT1 activation in αT3-1-myrAKT1 cells (Figures 4C and Additional file 7).

**Figure 5:**
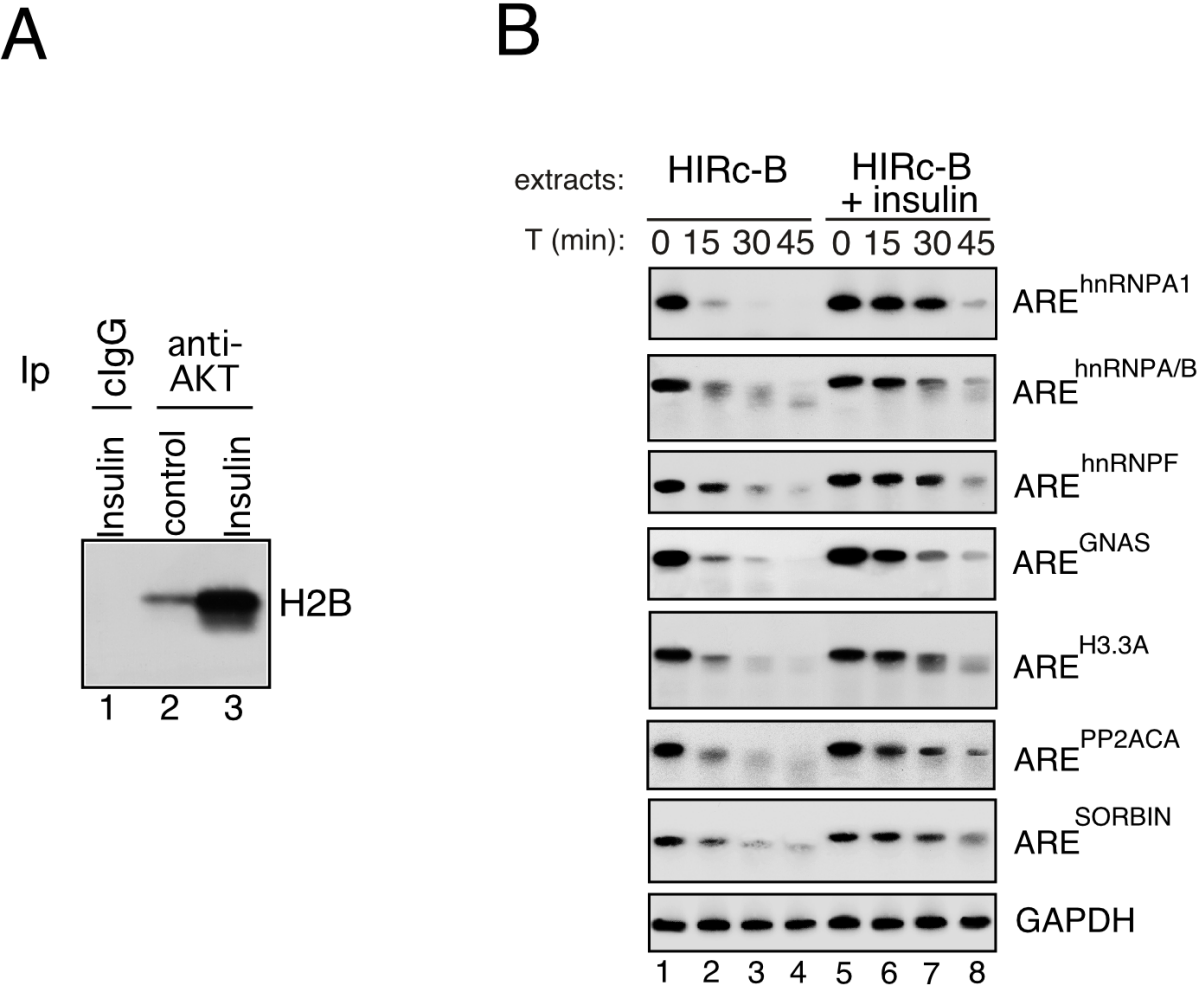
Insulin stabilizes a set of KSRP-interacting mRNAs. (A) Serum-starved HIRc-B cells were treated for 1 h with either PBS (control) or insulin (10^-6 ^M). Total extracts were immunoprecipitated with either anti-AKT antibody or control IgG (cIgG). Pellets were incubated (20 min at 30°C) with histone 2B (H2B) in kinase buffer in the presence of γ[^32^P]ATP under gentle shaking. Labeled proteins were separated by SDS-PAGE and detected by autoradiography. (B) In vitro RNA degradation assays using S100 extracts from either control or insulin (10^-6 ^M)-treated HIRc-B cells. Internally ^32^P-labeled, capped RNA substrates (see Additional file 4 for sequences) were incubated with S100 extracts for the indicated times and their decay analyzed as described in Methods.

Since PI3K-AKT signaling is known to be physiologically activated by insulin treatment [[12], and literature cited therein], we investigated the effect of insulin treatment on the decay rates of KSRP target transcripts. Insulin treatment of insulin receptor overexpressing HIRc-B cells produced a strong activation of immunoprecipitated AKT activity (Figure 5A) and caused stabilization of KSRP targets in vitro (Figure 5B).

Notably, both the t1/2 and steady-state levels of some of the KSRP targets (Table 2), as exemplified by PTMA, were not affected by PI3K-AKT activation although increased by KSRP knock-down in αT3-1 cells (Additional file 8).

Altogether, these data indicate that activation of PI3K-AKT signaling increased both the t1/2 and the steady-state levels of a subset of KSRP target transcripts.

## Discussion

Here we report that KSRP controls the half-life and the steady-state levels of a set of unanticipated labile mRNAs in αT3-1 cells. The expression and the stability of the majority of these KSRP target transcripts is increased upon activation of PI3K-AKT signaling. Furthermore, we show that KSRP forms a ribonucleoprotein complex together with its target transcripts and the RNA binding protein hnRNPA1.

Recently, we have shown that activation of PI3K-AKT pathway induces KSRP-controlled regulation of β-catenin mRNA in αT3-1 cells [12]. We hypothesized that PI3K-AKT activation could prolong the t1/2 of ARE-containing mRNAs additional to β-catenin by targeting KSRP.

In order to identify transcripts whose t1/2 and steady-state levels are controlled by KSRP and respond to PI3K-AKT activation, we performed a comparative analysis of the AU-rich transcriptome of αT3-1 cells focusing our attention onto KSRP target transcripts which are overrepresented in cells expressing a constitutively active AKT1. Microarray-based methods have been successfully used to study global patterns of transcript decay and comprehensively identify targets of RNA-binding proteins thus providing unique insights into gene expression programs [17,20-25]. We identified a set of mRNAs that interact with KSRP and whose t1/2 and steady-state levels are consistently increased by either KSRP knock-down or PI3K-AKT activation. Among these transcripts, three encode RNA binding proteins mainly implicated in pre-mRNA splicing events (hnRNPA1, hnRNPF, and hnRNPA/B), three encode signaling molecules (Gsα, PP2ACA, SORBIN), and one encodes the replication-independent histone H3.3A. To our best knowledge, none of these transcripts has been yet reported to undergo posttranscriptional control of its expression through regulation of mRNA decay rates.

Our present data (see Additional file 2) together with our previous observations [7,10-12] indicate that KSRP interacts with a rather broad array of ARE-like sequences. The criteria used by KSRP to recognize its RNA targets remain still unknown, and to date there are no reports that provide an explanation for its target recognition at the molecular level. Our unpublished structural studies on KSRP domains (M.F. Garcia-Mayoral et al., submitted) show a modularity of the interaction between K-homology (KH) domains 3 and 4 that can increase the adaptability to different RNA sequences/structures thus providing a possible explanation for the ability of KSRP to recognize highly heterogeneous RNA targets. These data indicate that KH3 and KH4 can adapt to different AU-rich sequences within the ARE without being limited by a rigid, pre-existing protein-protein interaction (M.F. Garcia-Mayoral et al., submitted). This provides the protein with a flexible recognition unit than can adapt to different RNA sequences and can mediate interactions in the structural environment of different 3'UTRs.

PI3K-AKT signaling has been reported to cause phosphorylation and activation of the SR-family members of splicing factors [26,27]. Interestingly, hnRNPA1 has been shown to antagonize the splicing activity of SR proteins [28]. Increased expression of hnRNPA1 could be viewed as a mean by which PI3K-AKT signaling finely modulates select splicing events. Intriguingly, hnRNPA1 transcript interacts with KSRP in the context of a complex that includes hnRNPA1 protein, thus suggesting the existence of an auto-regulatory loop.

GNAS gene encodes the Gsα that is required for hormone-stimulated cAMP generation [29]. Recently, Chen et al. demonstrated that GNAS gene deletion causes increased insulin sensitivity targeting AKT [30]. Our data allow the hypothesis that AKT could, in turn, regulate Gsα expression and activity operating a negative feed back-control on insulin responsiveness.

Protein phosphatase 2A (PP2A) comprises a family of serine/threonine phosphatases, whose minimal component is a well conserved catalytic subunit [reviewed in [31]]. PP2A plays a prominent role in cell cycle regulation, cell morphology and development [31]. We have recently shown that PI3K-AKT activation increases the expression of β-catenin by prolonging its mRNA t(1/2) through functional inactivation of KSRP. Intriguingly, PP2A can de-phosphorylate β-catenin thus preventing its degradation and, therefore, it has been proposed as an activator of β-catenin signaling [32,33]. Therefore PI3K-AKT, inducing stabilization of the two KSRP target transcripts β-catenin and PP2ACA, could enhance the cellular levels of β-catenin protein operating a combinatorial positive control. On the other hand, either inhibition or disruption of PP2A complexes leads to AKT activation [31]. Therefore, it is possible that PP2ACA mRNA stabilization and enhanced expression could operate a negative feed-back on the effects of either exaggerate or inappropriate PI3K-AKT signaling activation [34]. A potential model for KSRP-mediated control of PI3K-AKT/β-catenin signaling is presented in Additional file 9.

SORBS1, the human gene that encodes SORBIN, was mapped to the locus which is a candidate region for insulin resistance found in Pima Indians [35]. CAP, the mouse homologue of SORBIN, is a cytoskeletal adaptor protein involved in modulating adhesion-mediated signaling events that lead to cell migration [36]. It has been shown that stable cell lines overexpressing CAP exhibit a reduced growth rate [37]. Recently, Katsanakis and Pillay showed that AKT phosphorylates the APS protein, a key factor in the signaling events that involve CAP [38]. Our data support the existence of an additional point of cross-talk between PI3K-AKT signaling and the SORBIN/CAP pathway in insulin signaling.

Variations in the expression of histone H3.3A, a cell cycle-independent replacement histone, during differentiation of murine erithroleukemia cells, has been hypothesized to depend on post-transcriptional regulatory events [39]. Although histone H3.3A expression regulation has not been reported to be controlled by PI3K-AKT signaling, it has been correlated to cell transformation and differentiation [40,41].

Further investigations will be necessary to elucidate the functional role, if any, of the coordinated decay control of the identified transcripts by PI3K-AKT signaling under different physiological and pathological conditions.

Our data indicate that KSRP interacts with AUF1p45 and hnRNPA1 in the cytoplasm of αT3-1 cells. Only one of the PI3K-AKT-regulated KSRP targets, hnRNPA/B, is very weakly immunoprecipitated by anti-AUF1 antibody (Figure 1C and Additional file 5). Conversely, anti-hnRNPA1 antibody efficiently immunoprecipitates KSRP target transcripts (Figure 1C). Both AUF1p45 and hnRNPA1 bind very weakly to the same RNAs in vitro (data not shown). hnRNPA1 has been implicated in many aspects of mRNA maturation, transport, turnover and in telomere and telomerase regulation [42,43]. Hamilton et al. [19] reported that hnRNPA1 interacts with ARE-containing mRNAs and suggested a role for this factor in ARE-mediated decay. Our findings allow to hypothesize that AUF1p45 and hnRNPA1 play some, yet unidentified, regulatory role in the ribonucleoprotein complex that includes KSRP and its target transcripts. We can hypothesize that, in response to certain stimuli, the two KSRP-interacting ARE-BPs could acquire high affinity binding for target mRNAs thus either potentiating or terminating the decay-promoting activity of KSRP on the same transcripts.

The mRNA stability promoting factor HuR interacts with KSRP target transcripts both in vitro (data not shown) and in intact cells (Figure 1C). We have previously reported that the balanced interaction of KSRP and HuR to common sets of transcripts could allow a fine tuning of mRNA decay regulation upon specific stimuli [10,11]. Similar results were obtained by Linker et al. [13]. Our present data further support the idea that complex interactions in the ARE-BP network are required to ensure accurate regulation of the t1/2 of select transcripts.

## Conclusion

In conclusion, we have identified several KSRP target mRNAs that are overrepresented upon activation of PI3K-AKT signaling. The interaction of KSRP with these transcripts was validated in vitro and in intact cells. Importantly, both KSRP knock-down and PI3K-AKT activation were found to increase the stability and the steady-state levels of these target mRNAs. Our findings provide comprehensive and valuable insight into the KSRP-containing ribonucleoprotein complexes that govern gene expression at the posttranscriptional level.

## Methods

### Yeast two hybrid screening

A cDNA fragment encoding amino acids 47–711 of human KSRP was cloned into pDBLeu vector (Invitrogen) and used as the bait. MaV203 yeast cells containing pDBLeu-KSRP constructs were tested for self-activation and the concentration of 3-Amino-1,2,3,-Triazole required to inhibit the basal endogenous expression of HIS3 gene was determined. A e12.5 mouse embryo head cDNA library was prepared using the pEXP-AD502 vector according to manufacturer's (Invitrogen) instructions. pDBLeu-KSRP-containing MaV203 yeast cells were transfected with the library and selected according to the activation of the three reporter genes HIS3, URA3 and LacZ according to the manufacturer's (Invitrogen) protocol.

### Isolation of KSRP-co-purifying RNAs

To isolate mRNAs co-purifying with KSRP, the SNAAP (isolation of specific nucleic acids associated with proteins) technique described by Trifillis et al. [16] was used with minor modifications. Briefly, both GST and GST-KSRP fusion protein were expressed in Escherichia coli BL21. Cells expressing either protein were resuspended in lysis buffer (20 mM HEPES, pH 7.6, 1.5 mM MgCl_2_, 10 mM KCl, 0.5 mM DTT, 1X Complete protease inhibitors (Roche)), disrupted by sonication, and insoluble material removed by centrifugation. To eliminate bacterial RNAs, the extract was treated with 200 U/ml micrococcal nuclease (GE Healthcare) in the presence of 1 mM CaCl_2 _at 30 C for 20 min, and the reaction was stopped with the addition of 5 mM ethylene glycol bis(2-aminoethyl ether)-N,N,N'N'-tetraacetic acid (EGTA). Approximately 4 mg of either GST or GST-KSRP were bound to 1 ml GST-beads in a total volume of 5 ml in RNA binding buffer (RBB; 10 mM Hepes, pH 7.6, 3.0 mM MgCl_2_, 100 mM KCl, 2 mM DTT, 5% glycerol, 0.5% Triton X-100, 1X Complete) at 4°C for 1 h. Unbound proteins were removed with ten 5-ml washes in RBB. The washed beads were resuspended in 5 ml of RBB containing 50 μg/ml heparin (Calbiochem) and 200 U/ml RNasin (Promega). Fifty mg of cytoplasmic S100 extracts from αT3-1 cells were precleared with 2 ml of glutathione Sepharose slurry (extensively washed in RBB) to remove background RNAs that bind to the glutathione Sepharose beads. Incubation of the precleared S100 extracts to the above described washed beads was carried out at 4°C for 1 h under rotation, followed by six 5-ml washes in RBB. The RNA was then extracted with phenol/chloroform (1:1) and chloroform, ethanol precipitated with GlycoBlue (Ambion), and washed with 70% EtOH. The dried RNA was resuspended in 50 μl DEPC-treated H_2_O.

### Microarray hybridization and computational analysis of AREs

The glass microarrays contained cDNA probes representing more than 3000 ARE-cDNAs and control clones (their identities were obtained from AU-rich element-containing mRNA database ARED 3.0 [2]). The microarrays were hybridized with cDNA generated from total RNA (15 μg) and labeled with either Cy5 or Cy3 (control). The utilized hybridization protocol (Genisphere kit, Genisphere, Inc., Hatfield, PA) eliminated the possibility of signal contribution from genomic DNA [15]. cDNA microarrays scanning, pre-processing, filtering of erroneous signals, and normalization were performed as described in [15].

314 mRNAs enriched by at least 1.8-fold in KSRP-bound RNA samples (KSRP targets) and 314 mRNAs that were not enriched in KSRP-bound RNA samples (non-KSRP targets) were extracted from the microarray data. The sequences of the 3' UTR of both groups were used as input for the MotifSampler algorithm. The MotifSampler algorithms finds over-represented motifs in sequence regions using Gibbs sampling that has been successfully applied for both promoter and unstranslated regions [44]. This strategy has been applied previously [45].

### Cells, transfections

Murine αT3-1 pituitary cells and rat HIRc-B fibroblasts were cultured in DMEM plus 10% FBS. αT3-1 cell transfections were performed using Lipofectamine Plus (Invitrogen), G418 (Invitrogen) was used at 500 μg/ml for selection. Cell pools of transfectants were used for experiments. Both mock-αT3-1 and αT3-1-myrAKT1 cells were starved in DMEM plus 0.5% FBS for 16 hrs prior to experiments. HIRc-B cells were starved in DMEM plus 0.1% FBS for 16 hrs prior to experiments or further treatments.

### shRNA-mediated KSRP knock-down

pSUPER-Puro-shKSRP was previously described [11]. αT3-1 cells were transfected using Lipofectamine Plus (Invitrogen). Transfectant pools were selected with 0.3 μg/ml puromycin (Sigma).

### Recombinant proteins and antibodies

Affinity-purified human KSRP, expressed using the Baculovirus system, was described in Briata et al. [11]. cDNA fragments encoding the entire coding sequence of human hnRNPA1, the entire coding sequence of murine AUF1p45, and nt. 202–2136 of human KSRP were cloned into the pGEX6 to generate GST-A1, GST-AUF1p45, and GST-KSRP respectively. E. Coli-expressed GST-A1 protein was digested by Prescission protease (GE Healthcare) according to manufacturer's instructions. Anti-KSRP rabbit polyclonal antibody was previously [7] described. Anti-AUF1 and anti-hnRNP-A1 monoclonal antibodies were a kind gift from Dr. Gideon Dreyfuss. Anti-TTP (rabbit polyclonal H-120) was from Santa Cruz. Anti-α-tubulin, and anti HuR (3A2) monoclonal antibodies were from Sigma and Santa Cruz, respectively.

### RNA in vitro degradation and UV crosslinking

^32^P-labeled RNAs were synthesized and used as substrates for in vitro degradation assays as reported [46]. UV-crosslinking experiments were performed as described [46].

### Immunoprecipitation of ribonucleoprotein complexes

Ribonucleoprotein complexes were immunoprecipitated from αT3-1 cell lysates as previously described [46]. Total RNA, extracted from either immunocomplexes or total cell lysates (input) was subjected to RT-PCR reactions. Primers are listed in Additional file 10.

### In vitro kinase assays

Kinase assays were performed using AKT kinase activity immunoprecipitated from cell lysated and histone H2 (Roche) as the substrate. [γ-^32^P]ATP (3000 Ci/mmol) was from GE Healthcare.

### Semi-quantitative RT-PCR

Cells under different culture conditions were treated with 5 μg/ml actinomycin D, harvested at the indicated times, and total RNA was isolated using RNeasy mini kit (Qiagen) and treated with DNAseI (Promega) according to manufacturer's instructions. cDNA first strand was obtained with Transcriptor Reverse Transcriptase (Roche) using 250 ng of total RNA and oligo-dT primer. PCR reactions were performed using the sequence-specific primers listed in Table 2 of the Additional Data. β2-microglobulin was used as an internal control for normalizing transcripts levels measured by RT-PCR. To optimize RT-PCR, preliminary dose-response experiments were performed to determine the range of first strand cDNA concentrations at which PCR amplification was linear for each target molecule essentially as reported in Briata et al. [11]. For each species of RNA analyzed, the amount of RT-PCR product (measured as densitometric units) was plotted against the input of first strand cDNA.

## Abbreviations

ARE, AU-rich element;

ARE-BP, ARE binding protein;

β2-MG, β2-microglobulin;

KH, K-homology;

myrAKT1, myristylated form of AKT1;

PI3K, phosphatidylinositol 3-kinase;

PP2A, Protein phosphatase 2A;

t1/2, half-life.

## Authors' contributions

TR, MT, and MP performed the experiments; GC contributed analysis tools; C-YC contributed reagents and discussed data; LA-H performed experiments; KSAK performed experiments, acquired and analyzed data, reviewed the manuscript; PB and RG conceived, designed and performed the experiments, analyzed the data, and wrote the manuscript. all authors read and approved the final manuscript. PB and RG equally contributed to this work.

## Supplementary Material

Additional file 1 Features of cDNAs probes present in the AU-rich element based microarrays. list of the features of cDNA sequences present in the AU-rich element based microarrays. Click here for file

Additional file 2 ARE-like motifs are prevalently represented among KSRP target transcripts when compared to non-KSRP targets. the table provides consensus motifs for KSRP target transcripts as derived from bioinformatics analysis of AU-rich element based microarrays screenings. Click here for file

Additional file 3 Distribution of average Cy5/Cy3 fluorescence ratios from two independent microarray hybridizations. 1.5 log_2 _has been chosen as the threshold applied for defining 80 target genes (the inset shows a magnification of the enriched region). Data represent average Cy5/Cy3 fluorescence ratios from two independent hybridizations of the AU-rich element based microarrays. Click here for file

Additional file 4 Sequence of the ARE-containing 3'UTR regions of KSRP target transcripts cloned into pCY vector. Canonical ARE pentamers are highlighted in yellow while U-rich stretches are underlied. the files provides the sequence of the 3' UTRs of KSRP target transcripts. Click here for file

Additional file 5 AUF1 interacts very weakly with PTMA and hnRNPA/B mRNAs in αT3-1 cells. The figure shows representative immunoprecipitation experiments of AUF1-containing ribonucleoprotein complexes containing either PTMA or hnRNPA/B mRNAs. Click here for file

Additional file 6 KSRP knock-down prolongs the t(1/2) of KSRP target transcripts. Half-lives are expressed in minutes and were calculated on the basis of data presented in Figure 3C. The table shows the half-lives (in minutes) of KSRP target transcripts calculated on the basis of diagrams presented in Figure 3C. Data for both mock-transfected and shKSRP-transfected cells are presented. Click here for file

Additional file 7 PI3K-AKT signaling prolongs the t(1/2) of a set of KSRP-interacting mRNAs. Half-lives are expressed in minutes and were calculated on the basis of data presented in Figure 4C. The table shows the half-lives (in minutes) of KSRP target transcripts calculated on the basis of diagrams presented in Figure 4C. Data for both mock-transfected and myrAKT1-transfected cells are presented. Click here for file

Additional file 8 Both the stability and the steady-state levels of prothymosin α (PTMA) mRNA are regulated by KSRP while are unaffected by AKT1 activation. The Figure shows that KSRP can regulate by itself both the stability and the steady-state levels of PTMA while these parameters are not affected by activation of AKT1 in the same cells. This suggests that not all KSRP target transcripts are controlled by PI3K-AKT signaling. Click here for file

Additional file 9 A working hypothesis for KSRP-mediated stabilization of PP2ACA mRNA in response to PI3K-AKT signaling activation. The cartoon presents a speculation on the potential interplays existing in the PI3K-AKT signaling pathway through the intervention of KSRP. Click here for file

Additional file 10 Primers used for RT-PCR reactions. The table shows a list of the transcript-specific primers used in RT-PCR reactions in order to analyze the expression of KSRP target transcripts. Click here for file

## Figures and Tables

**Table 1:** Transcripts whose levels are increased by at least 2 fold in αT3-myrAKT1 when compared with mock-αT3. Transcripts identified as KSRP targets (see Table 3) are typed in bold.

	Transcript name	Accession number	Protein function	Fold increase
1	CDP-diacylglycerol-inositol 3-phosphatidyltransferase (phosphatidylinositol synthase)	NM_006319	Catalyzes the biosynthesis of phosphatidylinositol.	3.74
2	SNAP91, synaptosomal-associated protein, 91 kDa homolog	NM_014841	Component of clathrin-coated vescicles.	3.71
3	Solute carrier organic anion transporter family, member 1C1	NM_017435.2	Mediates the Na(+)-independent high affinity transport of organic anions such as the thyroid hormones thyroxine (T4) and rT3.	3.39
4	**Heterogeneous nuclear ribonucleoprotein F (hnRNPF)**	NM_004966	RNA binding protein, splicing.	3.20
5	Fibroblast growth factor 5	NM_004464	Oncogene, can transform NIH 3T3 cells.	3.20
6	Tankyrase, TRF1-interacting ankyrin-related ADP-ribose polymerase 2	NM_025235.2	Involved in the regulation of telomere length.	3.15
7	Fibroblast growth factor 19	NM_005117	Has a role in brain development, overexpressed in colon adenocarcinoma cell line.	3.12
8	**Heterogeneous nuclear ribonucleoprotein A/B (hnRNPA/B)**	M65028	RNA binding protein.	3.11
9	Microtubule-associated protein RP/EB family member 1	NM_012325	Component of the microtubule cytoskeleton.	3.10
10	Protocadherin beta 9	NM_019119	Calcium-dependent cell-adhesion protein.	3.00
11	**H3 histone, family 3A (H3.3A)**	NM_002107.3	Replacement histone, replication independent protein.	2.96
12	Thyroid hormone receptor interactor 4	NM_016213	Transcriptional coactivator of nuclear receptors.	2.90
13	ELL associated factor 2	NM_018456	Transcriptional transactivator of ELL and ELL2 elongation activities.	2.87
14	RUN and SH3 domain containing 1	NM_014328	Signaling adapter.	2.86
15	Cytochrome c oxidase subunit VIIc	NM_001867	Component of cytochrome c oxidase.	2.80
16	Zinc finger protein 192	NM_006298	Transcriptional regulator.	2.68
17	Notch homolog 3	NM_000435	Forms a transcriptional activator complex.	2.66
18	PHD finger protein 12	NM_001033561	Transcriptional repressor.	2.65
19	**GNAS complex locus (Gsα)**	NM_000516	Guanine nucleotide-binding protein.	2.60
20	Nascent-polypeptide-associated complex alpha polypeptide	NM_005594	Prevents inappropriate targeting of non-secretory polypeptides to the endoplasmic reticulum.	2.58
21	Protein phosphatase 1, catalytic subunit, beta isoform	NM_002709	Ser/Thr phosphatase, essential for cell division.	2.55
22	**Sorbin and SH3 domain containing 1 (SORBIN)**	NM_001034955	Involved in insulin receptor signaling.	2.51
23	Phosducin-like	NM_005388	G protein modulator.	2.51
24	Immunoglobulin mu-binding protein 2	NM_002180.1	DNA binding protein.	2.50
25	ornithine decarboxylase antizyme 1	NM_004152	Destabilizes and promotes degradation of ornithine decarboxylase.	2.49
26	Septin 5	NM_002688	Involved in cytokinesis.	2.44
27	epidermal growth factor	NM_001963.2	Growth factor.	2.40
28	Fibrosin 1	NM_022452	Fibrogenic lymphokine.	2.31
29	Death-associated protein kinase 1	NM_004938	Pro-apoptotic calcium/calmodulin-dependent serine/threonine kinase.	2.29
30	Caveolin 2	NM_001233	Scaffolding protein within caveolar membranes. Interacts directly with G-protein alpha subunits and can functionally regulate their activity.	2.28
31	Dual specificity protein phosphatase 4	NM_001394	Regulates mitogenic signal transduction by dephosphorylating both Thr and Tyr residues on MAP kinases ERK1 and ERK2.	2.14
32	Brix domain-containing protein 2	NM_018321	Biogenesis of the 60S ribosomal subunit.	2.07
33	Catenin beta (CTNNB) *	NM_007614.2	Wnt signaling, cell transformation	2.05
34	**Protein phosphatase 2 (formerly 2A), catalytic subunit, alpha isoform (PP2ACA)**	NM_002715	Dephosphorylates several Ser/Thr kinases.	2.02
35	**Heterogeneous nuclear ribonucleoprotein A1 (hnRNPA1)**	NM_002136	RNA binding protein.	2.00

*The regulation of Catenin beta by PI3K-AKT signaling has been described elsewhere [12].

**Table 2:** Transcripts whose levels are increased by at least 3 fold upon GST-KSRP chromatography when compared with control GST chromatography.

Transcript name	Accession number	Fold enrichment	3'UTR features	Protein functions
RAVER2	NM_018211	8.2	11 ARE pentamers	RNA-binding protein
GNAS1	NM_000516	7.7	2 ARE pentamers	Guanine nucleotide -binding protein
Protein phosphatase 2A catalytic subunit alpha Isoform (PP2ACA)	NM_002715	6.9	6 ARE pentamers	Protein phosphatase
Sorbin and SH3 domain containing 1 (SORBIN)	NM_001034955	6.5	11 ARE pentamers	Insulin signaling
Histone 3.3A (H3.3A)	NM_002107.3	5.9	7 ARE pentamers	Nucleosome formation
hnRNPF	NM_004966	5.6	2 ARE pentamers	RNA-binding protein
Prothymosin alpha (28)	NM_002823	5.5	1 ARE pentamer	Transcription factor
hnRNPA2/B1	NM_002137	4.7	4 ARE pentamers	RNA-binding protein
ATP synthase mitochondrial F0 complex subunit G	NM_006476	4.1	4 ARE pentamers	Mitochondrial ATPase
hnRNPA/B	M65028	4.0	3 ARE pentamers	RNA-binding protein
hnRNPA1	NM_002136	4.0	2 ARE pentamers	RNA-binding protein
Ecotropic viral integration site 5	NM_005665	3.5	15 ARE pentamers	Oncogene
Catenin beta (CTNN) *	NM_007614.2	3.1	U-rich regions	Transcription/Signaling

*The interaction of Catenin beta mRNA with KSRP and its decay control have been described in detail elsewhere [12].

**Table 3:** Molecular partners of KSRP identified by two-hybrid screening.

***Protein name***	***Protein function***	***Accession number***
hnRNP-A1	RNA binding protein	P49312
hnRNP-A/B	RNA RNA binding protein	NP_034578
AUF1 p45	ARE binding protein	NP_031542
PABPN1	Poly(A) binding protein	XP_214172
eIF2B beta subunit	Translation factor	NP_663420
14-3-3ζ	Molecular chaperone	NP_035870
