# Supplementary material for: Identification of a set of KSRP target transcripts upregulated by PI3K-AKT signaling
Source: BMC Mol Biol. 2007 Apr 16;8:28. doi: 10.1186/1471-2199-8-28 (PMC1858702; doi:10.1186/1471-2199-8-28)
Supplement: Additional file 1 — Features of cDNAs probes present in the AU-rich element based microarrays. list of the features of cDNA sequences present in the AU-rich element based microarrays. [file 1471-2199-8-28-S1.pdf]

Additional file 1. Features of cDNAs probes present in the AU-rich element-based microarrays [15].

**Features of the 3'UTRs**	**number**
cDNAs from ARE-mRNAs	2591
cDNAs from non-ARE mRNAs	1217
cDNAs from housekeeping mRNAs	88
Total cDNAs from non-ARE mRNAs	1305
Total cDNAs	3896
