# Supplementary material for: Identification of a set of KSRP target transcripts upregulated by PI3K-AKT signaling
Source: BMC Mol Biol. 2007 Apr 16;8:28. doi: 10.1186/1471-2199-8-28 (PMC1858702; doi:10.1186/1471-2199-8-28)
Supplement: Additional file 2 — ARE-like motifs are prevalently represented among KSRP target transcripts when compared to non-KSRP targets. the table provides consensus motifs for KSRP target transcripts as derived from bioinformatics analysis of AU-rich element based microarrays screenings. [file 1471-2199-8-28-S2.pdf]

Additional file 2.

ARE-like motifs are prevalently represented among KSRP target transcripts when compared to non-KSRP targets.

KSRP targets[1]				Non-KSRP targets[2]				
Consensus	No.[3]	%[4]	[5]LL1	Consensus	No.	%	LL2	LL1/LL2
**UAUUUAUw**	235	75%	1898	**UUUAUUUU**	85	27%	662	2.9
**UwUAUUUw**	191	76%	1760	**UAUwUAUw**	83	26%	667	2.6
**nUGUrUrU**	177	61%	1376	**sUGUsUGU**	62	19%	494	2.8
**CnsCyUCC**	170	54%	1412	**CnsCCUCC**	69	22%	566	2.5

[1]KSRP mRNAs targets (n=314) whose expression was up-regulated by more than 1.8-fold in KSRP-bound RNA samples as measured by microarray screening experiments. [2]Non-KSRP mRNAs targets (n=314) not enriched in KSRP-bound RNA samples. [3]Number of sequences found to harbor the indicated motif. [4]%: Percentage of sequences that harbor the motif divided by the total number of input sequences (n=314). [5]Log-likelihood ratio of the motif: is a measure of motif quality and depends on the strength of the motif and also on the total number of instances of the motif. The higher the number, the higher is the motif quality [44].
