# Supplementary figures and images for: Identification of a set of KSRP target transcripts upregulated by PI3K-AKT signaling
Source: BMC Mol Biol. 2007 Apr 16;8:28. doi: 10.1186/1471-2199-8-28 (PMC1858702; doi:10.1186/1471-2199-8-28)

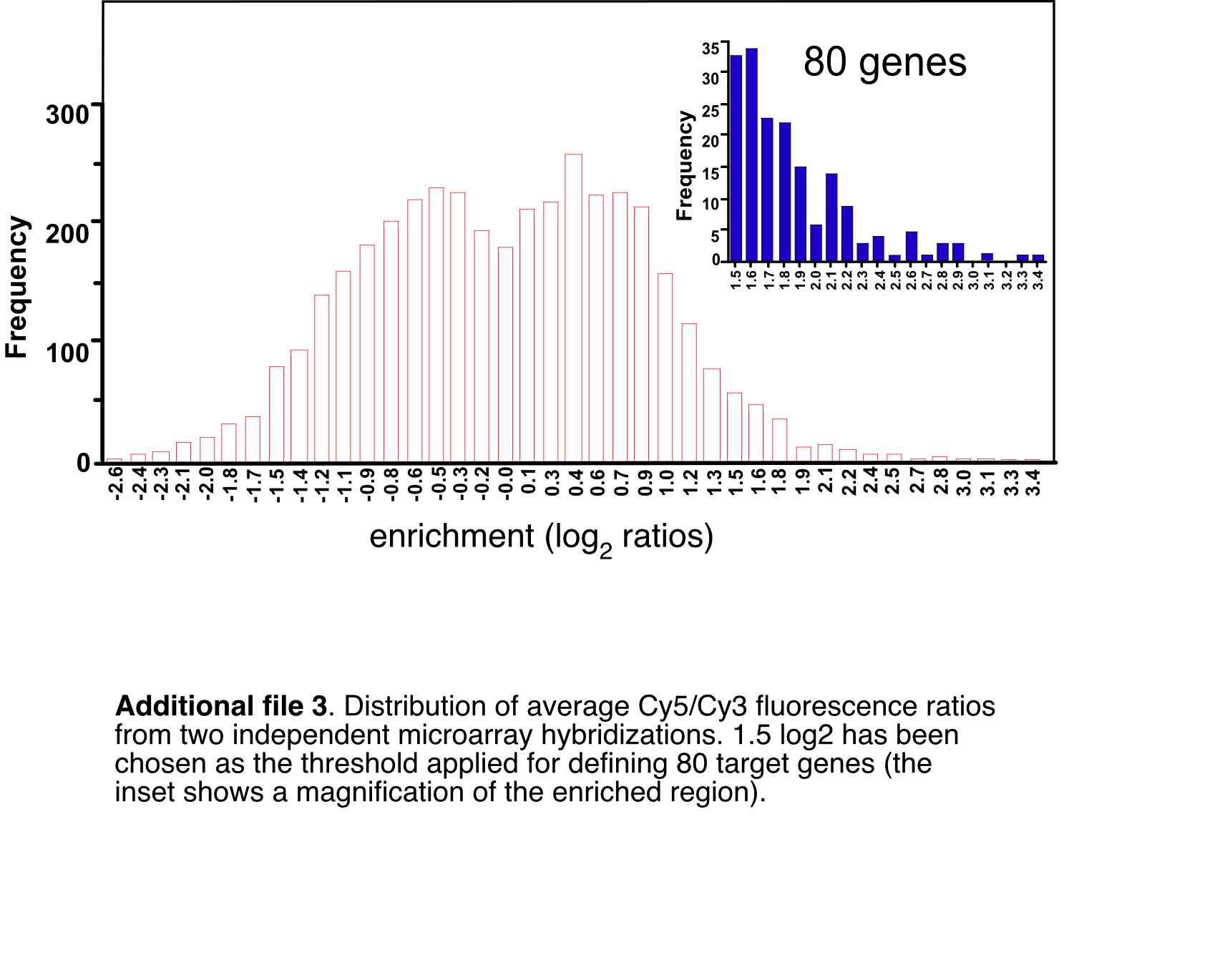

Supplement: Additional file 3 — Distribution of average Cy5/Cy3 fluorescence ratios from two independent microarray hybridizations. 1.5 log2 has been chosen as the threshold applied for defining 80 target genes (the inset shows a magnification of the enriched region). Data represent average Cy5/Cy3 fluorescence ratios from two independent hybridizations of the AU-rich element based microarrays. [file 1471-2199-8-28-S3.tiff]

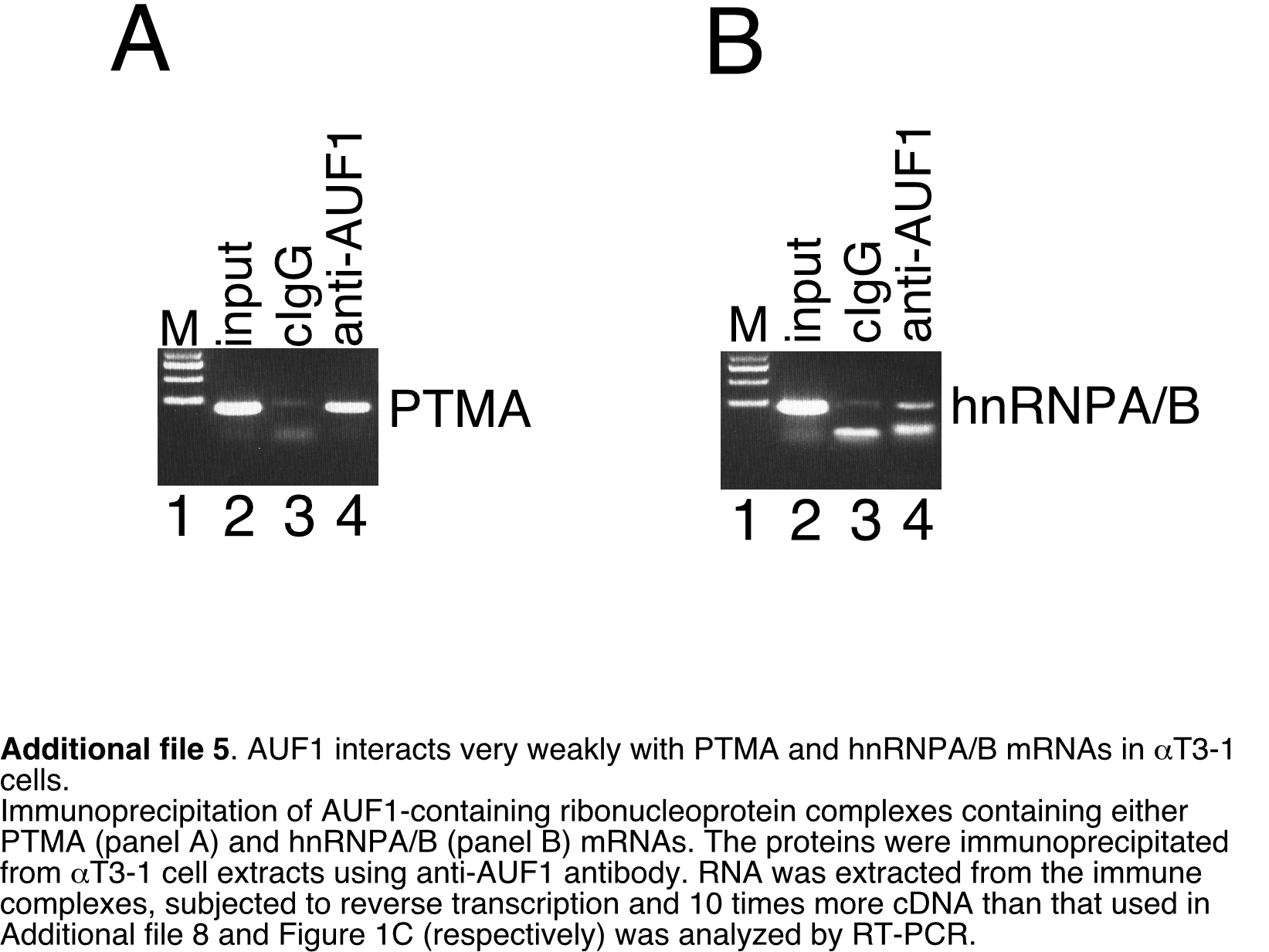

Supplement: Additional file 5 — AUF1 interacts very weakly with PTMA and hnRNPA/B mRNAs in αT3-1 cells. The figure shows representative immunoprecipitation experiments of AUF1-containing ribonucleoprotein complexes containing either PTMA or hnRNPA/B mRNAs. [file 1471-2199-8-28-S5.tiff]

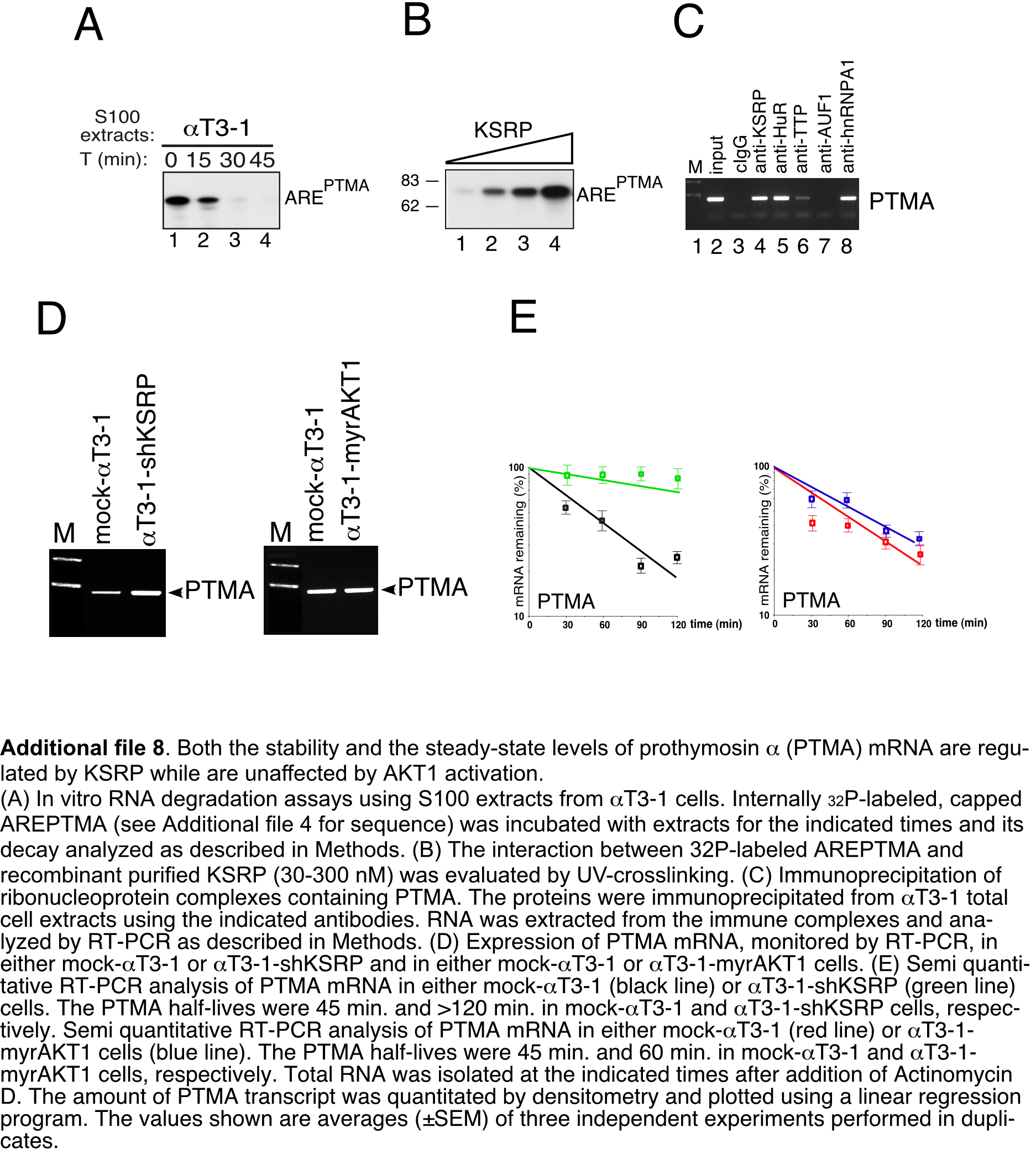

Supplement: Additional file 8 — Both the stability and the steady-state levels of prothymosin α (PTMA) mRNA are regulated by KSRP while are unaffected by AKT1 activation. The Figure shows that KSRP can regulate by itself both the stability and the steady-state levels of PTMA while these parameters are not affected by activation of AKT1 in the same cells. This suggests that not all KSRP target transcripts are controlled by PI3K-AKT signaling. [file 1471-2199-8-28-S8.tiff]

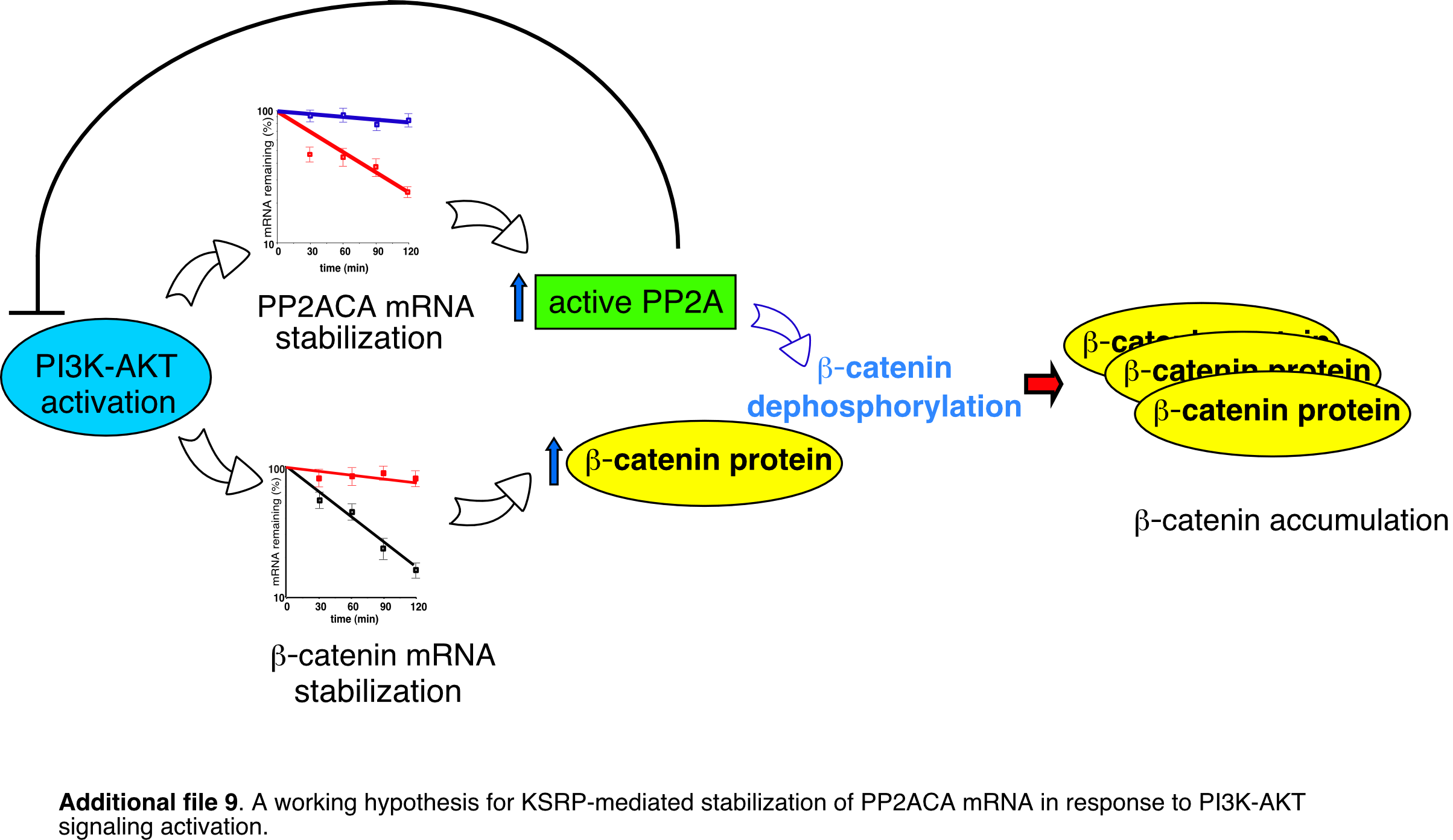

Supplement: Additional file 9 — A working hypothesis for KSRP-mediated stabilization of PP2ACA mRNA in response to PI3K-AKT signaling activation. The cartoon presents a speculation on the potential interplays existing in the PI3K-AKT signaling pathway through the intervention of KSRP. [file 1471-2199-8-28-S9.tiff]
