# Supplementary material for: Identification of a set of KSRP target transcripts upregulated by PI3K-AKT signaling
Source: BMC Mol Biol. 2007 Apr 16;8:28. doi: 10.1186/1471-2199-8-28 (PMC1858702; doi:10.1186/1471-2199-8-28)
Supplement: Additional file 4 — Sequence of the ARE-containing 3'UTR regions of KSRP target transcripts cloned into pCY vector. Canonical ARE pentamers are highlighted in yellow while U-rich stretches are underlied. the files provides the sequence of the 3' UTRs of KSRP target transcripts. [file 1471-2199-8-28-S4.pdf]

Additional file 4. Sequence of the ARE-containing 3'UTR regions of KSRP target transcripts cloned into pCY vector [18]. Canonical ARE pentamers are highlighted in yellow while U-rich stretches are underlied.

hnRNPA1

AACAGGUUAUUUUAGUUUCUGUUCUGUGGAAAGUGUAAAGCAUUCCAACA
AAGGGUUUUAAUGUAGAUUUUUUUUUUGCACCCCAUGCUGUUGAUUGC
UAAAUGUAACAGUCUGAUCGUGACGCUGAAUAAAUGUCUUUUUUUUAAUG
UGCUGUGUAAAGUUAGUCUACUCUUAAGCCAUCUUGGUAAAUUUCCCCAA
CAGUGUGAAGUUAGAAUUCCUUCAGGGUGAUGCCAGGUUCUAUUUGGAA
UUUAUAUACAACCUGCUUGGGUGGAGAAGCCA

hnRNPA/B

CACAUGCUUUGUUUGGAUAUGGAGUGAACACAAUUAUGUACCAAAUUUAA
CUUGGCAAACUUUCUAUUGCCUGUCCCAUGUGCAUCUUAUUUAAAAUUUC
CCCCAUGGAAAUCACUCUCC

hnRNPF

CAUUCUAGUUUAUAUUUUCAUUUAAAAUCCUUUAGGUUAAGUUUAAGCUU
UUUAAAAGUUAGUUUUGAGAAUUGAGACACAAUACUAAUACUGUAGGAAU
UGGUGAGGCCUUGACUUAAAACUUUCUUUGUACUGUGAUUUCCUUUUGG
GUGUAUUUUGCUAAGUGAAACUUGUUAAAUUUUUUGUUAACUAAAUUUUU
UUCUUAAAAUAAAGACUUUUUCACAAUGACUGGCACAGAUUACUCAGCAA
AAGAUAGCAAAACGGGUGGUUGAAGAUAAUUCAUUUUAAUCGUAAUGUAU
UUUAGUGUGAAUUUAAAAAUUUCAUACAUCA

GNAS

GGGAACCCCCAAAUUUAAUUAAAGCCUUAAGCACAAUUAAUUAAAAGUGAA
ACGUAAUUGUACAAGCAGUUAAUCACCCACCAUAGGGCAUGAUUAACAAA
GCAACCUUUCCCUUCCCCCGAGUGAUUUUGCGAAACCCCCUUUUCCCUU
CAGCUUGCUUAGAUGUUCCAAAUUUAGAAAGCUUAAGGCGGCCUACA

H3.3A

CAUUUCAUUCUCAAAAAAAAAAAAAAAUUUCUCUUCUUCCUGUUAUUGGU
AGUUCUGAACGUUAGAUAUUUUUUUUCCAUGGGGUCAAAAGGUACCUAAG
UAUAUGAUUGCGAGUGGAAAAAUAGGGGACAGAAAUCAGGUAUUGGCAG
UUUUUCCAUUUUCAUUUGUGUGUGAAUUUUUAAUAUAAAUGCGGAGACGU
AAAGCAUUAAUGCAAGUUAAAAUGUUUCAGUGAACAAGUUUCAGCGGUUC
AACUUUAUAAUAAUUAUAAAUAAACCUGUUAAAUUUUUCU

PP2ACA

AACACCUCGUGAAUACAAUUUAACUUCCAUUUAGCUAUAGCUUUACUCAG
CAUGACUGUAGAUAAGGAUAGCAGCAAACAAUCAUUGGAGCUUAAUGAAC
AUUUUUAAAAAUAAUUACCAAGGCCUCCCUUCUACUUGUGAGUUUUGAAA
UUGUUCUUUUUAUUUUCAGGGAUACCGUUUAAUUUAAUUAUAUGAUUUGU

SORBIN

UCAUUCUUUUCCCAUAUCCUUGGCUUUUAGAAAUGGUUACCUUCAGGACA
GUGCAGCUGCAUUUAUCAGAGCACUAUUGCUAAGUUUUCUUUUCUGGCU
UGUGUUUUUCUGGGACAGUUUAGAAUUGGGAGGCCUAUUCUCAUAGAAC
ACCAAAAAUGAUGUUCAGUGAUUCAUUUAACAUACACCAAUGUACUCUGG
CU

PTMA

AUUUGCAACAGGGGAGGAAAAAAGAACCAAAACUUCCAAGGCCCUGCUUU
UUUUCUUAAAAGUACUUUAAAAAGGAAAUUUGUUUGUAUUUUUUAUUUAC
AUUUUAUAUUUUUGUACAUAUUGUUAGGGUCAGCCAUUUUUAAUGAUCUC
GGAUGACCAAACCAGCCUUCGGAGCGUUCU
