# Supplementary material for: Identification of a set of KSRP target transcripts upregulated by PI3K-AKT signaling
Source: BMC Mol Biol. 2007 Apr 16;8:28. doi: 10.1186/1471-2199-8-28 (PMC1858702; doi:10.1186/1471-2199-8-28)
Supplement: Additional file 6 — KSRP knock-down prolongs the t(1/2) of KSRP target transcripts. Half-lives are expressed in minutes and were calculated on the basis of data presented in Figure 3C. The table shows the half-lives (in minutes) of KSRP target transcripts calculated on the basis of diagrams presented in Figure 3C. Data for both mock-transfected and shKSRP-transfected cells are presented. [file 1471-2199-8-28-S6.pdf]

Additional file 6. KSRP knock-down prolongs the t(1/2) of KSRP target transcripts. Half-lives are expressed in minutes and were calculated on the basis of data presented in Figure 3C.

Transcript	mock-αT3-1	αT3-1-shKSRP
hnRNPA1	40	>120
hnRNPA/B	65	>120
hnRNPF	60	>120
GNAS	60	>120
H3.3A	45	>120
PP2ACA	60	>120
Sorbin	40	>120
β2-MG	>120	>120
