# Supplementary material for: Identification of a set of KSRP target transcripts upregulated by PI3K-AKT signaling
Source: BMC Mol Biol. 2007 Apr 16;8:28. doi: 10.1186/1471-2199-8-28 (PMC1858702; doi:10.1186/1471-2199-8-28)
Supplement: Additional file 7 — PI3K-AKT signaling prolongs the t(1/2) of a set of KSRP-interacting mRNAs. Half-lives are expressed in minutes and were calculated on the basis of data presented in Figure 4C. The table shows the half-lives (in minutes) of KSRP target transcripts calculated on the basis of diagrams presented in Figure 4C. Data for both mock-transfected and myrAKT1-transfected cells are presented. [file 1471-2199-8-28-S7.doc]

**Additional file 7**. PI3K-AKT signaling prolongs the t(1/2) of a set of KSRP-interacting mRNAs. Half-lives are expressed in minutes and were calculated on the basis of data presented in Figure 4C.

| Transcript | mock-αT3-1 | αT3-1-myrAKT1 |
| --- | --- | --- |
| hnRNPA1 | 50 | >120 |
| hnRNPA/B | 70 | >120 |
| hnRNPF | 50 | >120 |
| GNAS | 50 | >120 |
| H3.3A | 55 | >120 |
| PP2ACA | 60 | >120 |
| Sorbin | 45 | >120 |
| β2-MG | >120 | >120 |
