# Supplementary material for: Identification of a set of KSRP target transcripts upregulated by PI3K-AKT signaling
Source: BMC Mol Biol. 2007 Apr 16;8:28. doi: 10.1186/1471-2199-8-28 (PMC1858702; doi:10.1186/1471-2199-8-28)
Supplement: Additional file 10 — Primers used for RT-PCR reactions. The table shows a list of the transcript-specific primers used in RT-PCR reactions in order to analyze the expression of KSRP target transcripts. [file 1471-2199-8-28-S10.pdf]

Additional file 10. Primers used for RT-PCR reactions.

Transcript	Forward primer	Reverse primer
hnRNPA1	5'—CTA ATT GTA TAA CAG GTT ATT T—3'	5'—CCA AGC AAC CAT AAA TAA ATT CCA—3'
hnRNPA/B	5'—CGC ACA CGC TTT GTT TGG ACG C—3'	5'—TGA TCT AGA ACA GGG TAA CCT GG—3'
hnRNPF	5'—CAC TTT GCA CCA TGA GTT TGT G—3'	5'—TAA CAT CAC TCT TAA GTA TAC TAC—3'
GNAS	5'—TAC GAG CTG CTC TAA GAA GGG A—3'	5'—TCT GTA GGC CGC CTT AAG CTT—3'
H3.3A	5'—TAA TGC AAG CAA AAT GTT TCA GTG—3'	5'—AAA CAC CAT TTA GTT GCC GTC A—3'
PP2ACA	5'—CAA TCA TGG AAC TTG ACG A—3'	5'—TTT GGA GTT ACT GTT GCT CTT—3'
SORBIN	5'—GAA GAC TAA AAA GCA CA—3'	5'—CAG CTC CCA GAA GC—3'
PTMA	5'—ACA ACA TGA ATT GGC AAC ATG G—3'	5'—ACA GAA AAC GCT CTG AAG GCT GG—3'
GM-CSF	5'—CTA CCA GAC ATA CTG CCC CCC—3'	5'—CTG TCC AAG CTG AGT CAG CG—3'
β2-MG	5'—GTC TTT CTG GTG CTT GTC TCA—3'	5'—GGC GTA TGT ATC AGT CTC AGT—3'
